# Pseudoaneurysm of the lingual artery as an unusual radiological finding of oropharyngeal carcinoma

**DOI:** 10.1259/bjrcr.20200063

**Published:** 2020-10-22

**Authors:** Irene Espallargas, Salvatore Marsico, Flavio Zuccarino, Sofia González-Ortiz, José María Maiques, Jaume Capellades, Santiago Medrano

**Affiliations:** 1 Department of Radiology, Hospital del Mar, Passeig Marítim 25, 08003 Barcelona, Spain; 2 Department of Radiology, Hospital Germans Trias i Pujol, Carretera del Canyet, s/n, 08916 Badalona, Barcelona, Spain; 3 Department of Radiology, Hospital Sant Joan de Déu, Passeig de Sant Joan de Déu, 2, 08950 Esplugues de Llobregat, Barcelona, Spain

## Abstract

Pseudoaneurysms of the lingual artery are an extremely rare entity and often are consequence of neck surgery, trauma or inflammation (*e.g.,* due to chemoradiotherapy or odontogenic infection), and may cause life-threatening bleeding. To our knowledge, this is the first report of buccal bleeding secondary to the presence of a previously undiagnosed oropharyngeal carcinoma with an associated lingual artery pseudoaneurysm.

## Clinical presentation

An 89-year-old female with history of hypertension and hyperlipidaemia associated with ischaemic and valvular heart disease was referred to emergency department due to uncontrolled buccal bleeding and pharyngeal discomfort. The only reported risk factor was treatment with aspirin 100 mg/day. The patient had no previous history of oral bleeding. Upper GI endoscopy for oropharyngeal dysphagia had been performed 3 weeks before the onset with no pathological findings. Haemoptysis and haematemesis were ruled out by physical examination, evidencing a posterior left hemilingual flat lesion with a tongue depressor.

The patient’s haemoglobin decreased from 13.4 to 10.6 g/dl in no longer than 6 h. Blood pressure was 94/43 mm Hg, heart rate was 90 bpm.

## Imaging findings

Patient underwent baseline unenhanced CT of the neck, followed by CT angiography of the neck with bolus tracking technique (region of interest located at the aortic arch with a trigger threshold of 100 UH) and venous phase at 70 s, after an injection of 50 ml of non-ionic iodinated contrast media at a flow rate of 4 ml s^−1^, followed by a 25 ml saline flush, with the following parameters: tube voltage 140 kV, 340 mA, pitch = 0.984, rotation time = 0.5 s, field of view = 512×512 mm, 64×0.625-mm detector configuration and slice thickness = 1.25 mm.

**Figure 1.**
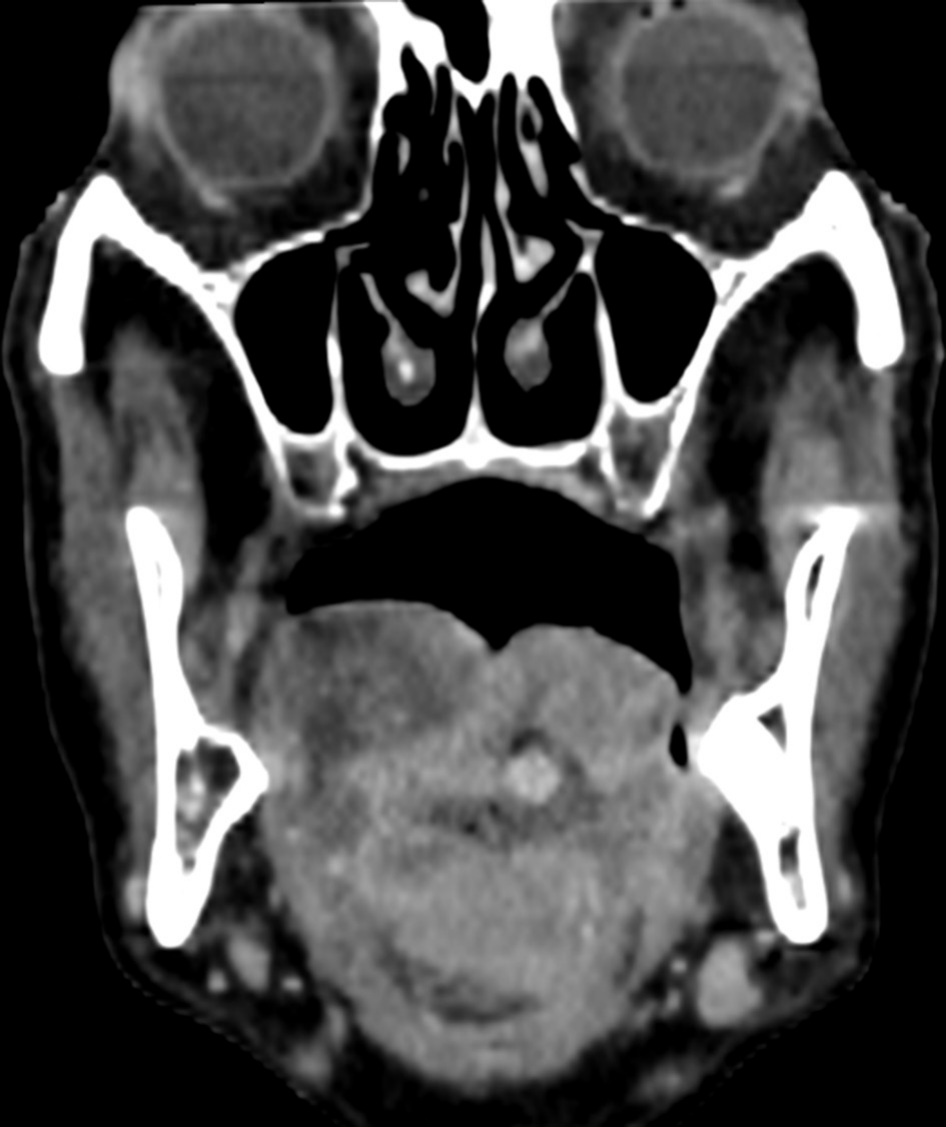
Coronal venous CT image, revealing the extension of the lesion to the two posterior thirds of the tongue and an enhancing pseudoaneurysm within the necrotic/ulcerative centre of the mass.

A large, infiltrating, ulcerative and heterogeneously enhancing mass located among the base of the tongue and the two posterior thirds of the oral tongue was depicted, affecting both sides of the middle line and being highly suspicious of oropharyngeal origin (Figure 1). It involved all of the extrinsic muscles of the tongue and reached the left epiglottic vallecula with a minimal infiltration of the left palatine tonsil.

There were no signs of extension to the pre-epiglottic space. Pterygopalatine fossa, pterygoid muscles and mandibular bone were also preserved.

**Figure 2.**
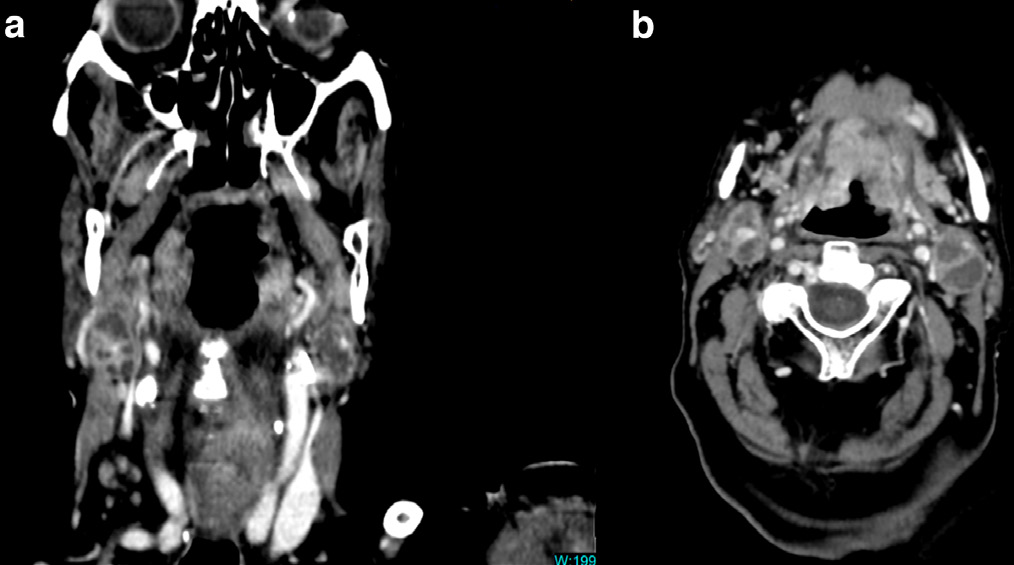
Level II A ill-defined bilateral necrotic lymphadenopathies in coronal (A) and transverse (B) venous phase CT images. Irregular capsular enhancement and obliteration of fat planes next to the sternocleidomastoid muscles suggest extranodal invasion.

Level II A bilateral necrotic lymphadenopathies were found. They measured less than 3 cm and showed signs of extranodal extension (Figure 2).

**Figure 3.**
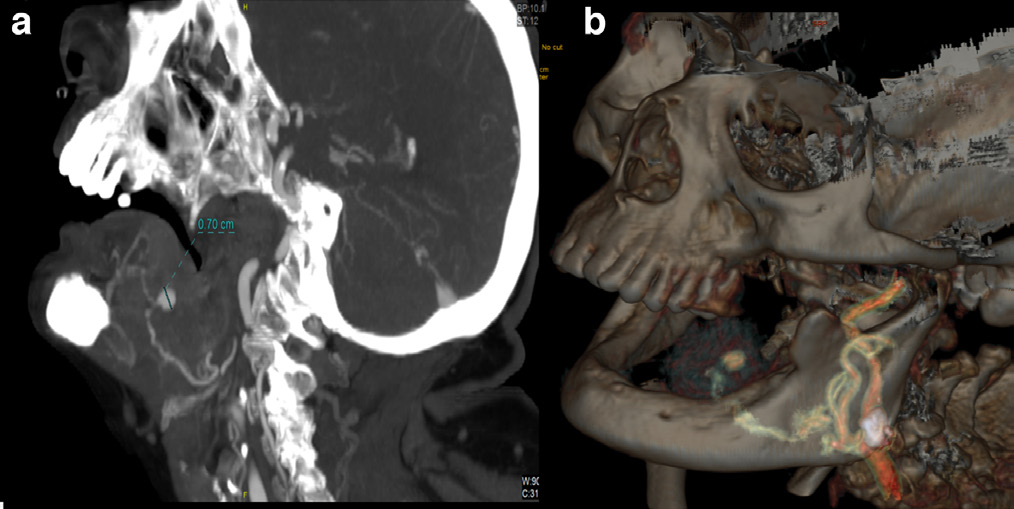
Sagittal MIP CTA (A) and 3D volume rendering (B) images depict a 7 mm aneurysmatic formation emerging from the lingual artery.

**Figure 4.**
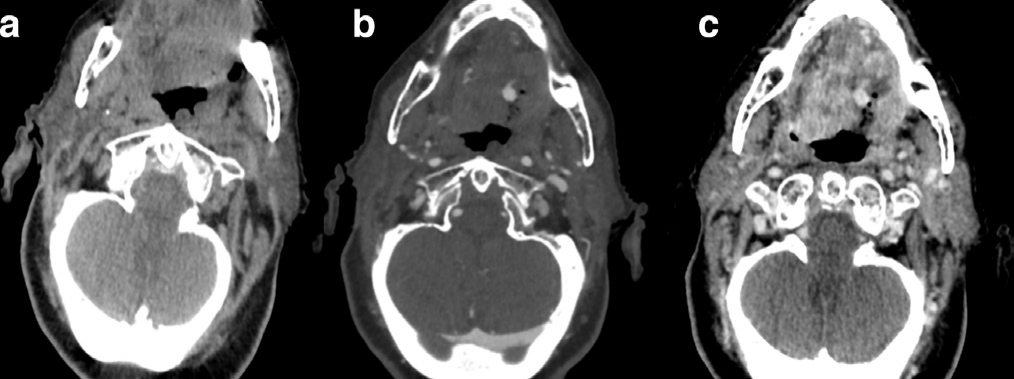
Transverse images of unenhanced (A), angiographic (B) and venous (C) CT phases show a large ulcerative mass at the base of the tongue with a pseudoaneurysm within, and no obvious contrast media extravasation.

CT angiography revealed a 7 mm pseudoaneurysm within the tumour, arising from the left lingual artery (Figures 3 and 4).

It was concluded that both the pseudoaneurysm and a T4aN3B (if HPV was negative) or T4aN2 (if positive) oropharyngeal carcinoma were the main source of the bleeding.

## Treatment

Subsequent to the CT diagnosis, treatment with transexamic acid and subcutaneous morphine was established. Due to age and comorbidities, the patient was not considered a candidate for emergency transarterial embolization.

## Outcome and follow-up

The patient was admitted to the oncology ward with progressive improvement of the bleeding and tolerance to oral food intake. However, 6 days after the CT scan, she presented major bleeding and desaturation leading to in-hospital death.

## Discussion

A false aneurysm or pseudoaneurysm is by defining a vessel haematoma in communication with the arterial lumen, contained by the adventitia or adjacent soft tissue. Its high intrinsic risk of rupture demands embolization in most cases.

Schechter et al^1^ reported that external carotid artery aneurysms were 2.2% of all cervical carotid aneurysms, while lingual artery aneurysms were particularly rare, although their prevalence is unclear. Iwai et al^2^ reported an incidence of 0.11%, with just one case out of 926 oral cancer patients. However, this diagnosis was performed incidentally and had no relation with the patient’s tongue carcinoma.

Although the majority of false aneurysms of the lingual artery have a traumatic or surgical origin (especially tonsillectomy),^3–5^ some cases have been related to radiofrequency, chemoradiation and infection.^6–8^ Idiopathic aneurysms have also been described.^2,9,10^ There are also few cases of already diagnosed oropharyngeal carcinomas that presented with bleeding due to lingual artery pseudoaneurysms.^7,11^

In the case reported, there were no previous CT scans to confirm that the pseudoaneurysm was not a pre-existent condition. However, there was no history of iatrogenesis or trauma, and the extent of the tumoral mass and its intimate relation with the pseudoaneurysm raised radiological suspicion of its origin in the described neoplasm.

The patient described was not deemed for further treatment, nevertheless there is a less invasive alternative to transarterial embolization, the US-guided percutaneous thrombin injection,^12,13^ although expert management is required and distal embolization can occur.

In the present case, we aim to draw attention to a potentially life-threatening condition developed within an oropharyngeal/oral cavity tumour that eventually led to patient’s death. Therefore, in patients with suspected oral cancer and active bleeding, CT angiography scan ought to be performed first to identify aneurysms or pseudoaneurysms, while a delayed phase should also be acquired to depict its anatomical relations, as mucosal enhancement improves the definition of the mass. However, if the patient is in severe hypovolaemic shock, endovascular treatment should not be delayed.^14^

## Learning points

1. Oropharyngeal carcinomas may present with pseudoaneurysm of the lingual artery, even before any treatment has been established.
2. CT angiography followed by a delayed phase may be the gold standard for depicting pseudoaneurysms in clinically stable patients.
3. Pseudoaneurysms of the lingual artery are potentially life-threatening and radiologist’s suspicion is essential to determine the CT protocol.
